# Supplementary material for: Developing and validating a multi-criteria decision analytic tool to assess the value of cancer clinical trials: evaluating cancer clinical trial value
Source: Cost Eff Resour Alloc. 2023 Nov 14;21:87. doi: 10.1186/s12962-023-00496-y (PMC10647033; doi:10.1186/s12962-023-00496-y)
Supplement: Supplementary file 4 — Supplementary Material 4 [file 12962_2023_496_MOESM4_ESM.docx]

# **Developing and validating a multi-criteria decision analytic tool to assess the value of cancer clinical trials**

## Value of cancer clinical trials

Piers Gillett^1^ (0000-0002-7361-2863), Robert K Mahar^1,2^ (0000-0002-3643-923X), Nancy R Tran^1^ (0000-0002-1530-1450), Mark Rosenthal^3,4^ (0000-0003-1152-6764) and Maarten IJzerman^1,3*^ (0000-0001-5788-5805)

1. Cancer Health Services Research Unit, Centre for Health Policy, Melbourne School of Population and Global Health, University of Melbourne, Australia
2. Biostatistics Unit, Centre for Epidemiology and Biostatistics, Melbourne School of Population and Global Health, University of Melbourne, Australia
3. Sir Peter MacCallum Department of Medical Oncology, University of Melbourne, Australia
4. Department of Medical Oncology, The Royal Melbourne Hospital, Australia

Corresponding author: Maarten IJzerman, University of Melbourne Centre for Cancer Research, 305 Grattan St, Melbourne 3000 Australia.

Email: [maarten.ijzerman@unimelb.edu.au](mailto:maarten.ijzerman@unimelb.edu.au)

Phone: +61385598585

**Word count:** 3765

Statements and Declarations

## Funding

This work was initiated at the request of and funded by the Victorian Comprehensive Cancer Centre (VCCC) to improve the outcomes and efficiencies of the clinical trial programs throughout member institutions.

## Competing interests

The authors declare that they have no competing interests.

Ethics Approval

Ethics approval for the study was provided by the Melbourne School of Population and Global Health Human Ethics Advisory Group at the University of Melbourne (ID 2056390.3).

Consent to participate

Informed consent was obtained from all individual participants included in the study.

Availability of data and materials

The datasets generated and analysed during the current study are not publicly available due to consent being specific to this project with no future use permitted. Data may be made available if consent for release can be reasonably obtained from study participants.

Author’s contributions

PG was the lead investigator on this project and primarily supervised by RK and MIJ. All authors contributed to the conceptual design, data collection, analysis and manuscript creation in line with their authorship position. The final manuscript was read and approved by all authors.

## **Acknowledgements**

We would like to thank members of the steering committee Anne Woolett, Courtney Thornely, Grant McArthur, Christie Allan, Dayna Swiatek, Meredith Layton, Phil Kiossoglou, Clare Slaney and Eva Segelov for providing feedback and guidance throughout the project. We would also like to thank Anneke Grobler, Jo Cockwell, Paul Baden, Paul Fennessy, Suzanne Hasthorpe, Simon Montgomery, Sophie Broughton, Keith Donahoe and Leslie Leckie who provided feedback and their perspective on proposed criteria.

We would like to thank Atara Posner, Colin Wood, Grace Chazan, Sharon Carvalho, Michelle Tew and Sophy Athan for participating in the partial value function elicitation.

We would also like to thank all those who responded to the survey.

## **Abstract**

**Background**:

Demonstrating safety and efficacy of new medical treatments requires clinical trials but clinical trials are costly and may not provide value proportionate to their costs. As most health systems have limited resources, it is therefore important to identify the trials with the highest value. Tools exist to assess elements of a clinical trial such as statistical validity but are not wholistic in their valuation of a clinical trial. This study aims to develop a measure of clinical trials value and provide an online tool for clinical trial prioritisation.

**Methods**: A search of the academic and grey literature and stakeholder consultation was undertaken to identify a set of criteria to aid clinical trial valuation using multi-criteria decision analysis. Swing weighting and ranking exercises were used to calculate appropriate weights of each of the included criteria and to estimate the partial-value function for each underlying metric. The set of criteria and their respective weights were applied to the results of six different clinical trials to calculate their value.

**Results**: Seven criteria were identified: ‘unmet need’, ‘size of target population’, ‘eligible participants can access the trial’, ‘patient outcomes’, ‘total trial cost’, ‘academic impact’ and ‘use of trial results’. The survey had 80 complete sets of responses (51% response rate). A trial designed to address an ‘Unmet Need’ was most commonly ranked as the most important with a weight of 24.4%, followed by trials demonstrating improved ‘Patient Outcomes’ with a weight of 21.2%. The value calculated for each trial allowed for their clear delineation and thus a final value ranking for each of the six trials.

**Conclusion**: We confirmed that the use of the decision tool for valuing clinical trials is feasible and that the results are face valid based on the evaluation of six trials. A proof-of-concept applying this tool to a larger set of trials with an external validation is currently underway.

## **Keywords**

Multi-criteria decision analysis, clinical trials, cancer, decision tool, decision analysis, prioritisation, portfolio

**1 Introduction**

Clinical trials play a key role in establishing safety and efficacy of new modes of medical care. Beyond this, trials also generate externalities that accrue to different stakeholders, each of whom may value a trial in ways that go beyond safety or efficacy. Minimising the uncertainty of treatment efficacy benefits future patients. Additionally, greater certainty of a treatment’s benefits is a requirement of health economic models that justify or redirect resources to areas of need and is essential for market authorisation and policy making (1).

Clinical trials are often costly, yet it remains difficult to establish whether a trial produces value commensurate with their cost. The respective average cost of phase I, II, and III trials of investigational compounds globally reached approximately $25.3M, $58.6M and $255.4M in 2013 U.S. dollars (2). In Australia, the cost of early-stage/phase I trials is also high although being approximately 28% lower than the U.S. (3). This leads to the presumption that, in a society with limited resources, only trials that pose the highest value for society should be prioritised (4). The American Society of Clinical Oncology (ASCO) (5) and the European Society of Medical Oncology (ESMO) (6) each provide frameworks to quantify gains of new cancer treatments based on patient survival, treatment toxicity and quality of life as determined in a clinical trial. While both scales have value, the outputs of the ASCO and ESMO frameworks are not well correlated. Moreover, a negative correlation between ASCO medical benefit scores and monthly drug costs was reported (7). Some have found that most drugs enter the market without evidence of survival gain (8). With high trial costs and relatively few trials providing clinical results that meet the magnitude of clinical benefit (9), it is important to establish a value threshold inclusive of diverse stakeholder preferences and sufficient to change clinical practice in order to justify high costs of the trial in the first place.

Because trial results can be used to inform future research directions, inform policy, or prompt changes in clinical practice, trial conclusions must be scientifically robust. Efforts to address this have produced tools such as the Cochrane risk-of-bias tool (10), the Jadad score (11), a general tool assessing methodological quality of a trial (12), a tool for methodological strength of orthopaedic surgery-focused trials (13) and the Delphi List (14). Yet these tools tend to focus on relatively narrow definitions or a specific element of clinical trial value and none provide a broad measure of clinical trial value incorporating all stakeholder views.

Multi-criteria decision analysis (MCDA) is a method that enables users to assess conflicting criteria together to assist in decision making (15). Each MCDA criterion represents something that at least some stakeholders consider important in decision making. MCDA helps to jointly assess each criterion. MCDA is a general concept and can be used for a variety of decision problems, including supporting patient choices and portfolio management (16–18). Many different approaches have been proposed as MCDA, although many differences exist in how criteria are weighted and how overall value functions are estimated. For instance, some methods allow the decision maker to explicitly quantify the value of each decision based on each different criterion as opposed to a purely qualitative output (19). However, one feature that all methods have in common is that of selecting decision alternatives (different trials in our study), identifying criteria relevant for the decision, gauging the performance of the decision alternatives with respect to the criteria, weighting the criteria in terms of their importance and aggregating the weighted criteria into a single value (19). Examples of this approach can be seen in Chapple et al., 2020 (20) and Ahuja et al., 2023 (21). In this study we used recommended MCDA methods per the ISPOR TaskForce .(19)

In this study we implemented MCDA using swing weighting and ranking methods where responses from a range of representative stakeholders relevant for the design, conduct and results of clinical trials were collected. We recorded their opinions on which trial characteristics (herein referred to as ‘criteria’) of clinical trials encapsulated trial value and the relative value of each metric. In this study we will summarise = how we developed an MCDA-based decision tool to evaluate cancer clinical trials and then applied the tool to retrospectively evaluate a portfolio of cancer clinical trials.

## **2 Methods**

### 2.1 Step 1: Stakeholder selection

Consultation with relevant stakeholders was conducted at multiple stages throughout the development process. Stakeholders were identified through the professional networks of the authors and consumer advocate networks within the Victorian Comprehensive Cancer Centre (VCCC) alliance. Stakeholders were included to capture expertise from as many relevant domains as possible. These stakeholders included clinicians, statisticians, scientists, operations researchers, regulators, health economists, clinical trial unit managers, and consumer representatives. All stakeholders who responded to our invitation to participate were included. Any given stakeholder representative was only involved in one round of consultation so as not to overly influence results.

### 2.2 Step 2: Identifying criteria

A search through academic and grey literature was undertaken to establish a ‘starting set’ of criteria used to determine trial value. Two reports from RAND Europe, the first by Guthrie et al. (22) and the second by Deshpande et al. 2018 (20) were identified, from which were identified, from which an initial set of eight criteria were tentatively selected.

The first round of stakeholder consultation augmented the initial list of eight criteria. Interviewees were presented with the aims of our study and asked to suggest criteria they thought would “best represent clinical trial value”. Responses were either recorded manually or using Poll Everywhere, a web-based polling service (24). Stakeholders were then asked to rank how representative of trial value the augmented set of criteria were for each of clinical trial phases I, II and III respectively using Poll Everywhere.

The second round of stakeholder consultation considered the augmented set of criteria. The participating stakeholder representatives were presented with three real-world trials, each from a different phase and with different interventions and asked to nominate which they considered the most valuable. Attendees were given a 10-minute time limit and could ask questions throughout. Participants were then asked to identify the key metric that, should its value change significantly, would change their decision. This was then repeated for each of the criteria of the augmented set.

In the third round of stakeholder consultation, we conducted a series of face-to-face interviews focusing upon patient advocates and health regulators. These interviews were conducted as open discussions between the research team and participants. We presented the participants with a list of criteria established through the previous rounds of polling and interviews. Participants were asked to rank the criteria representing trial value from ‘most representative’ of value to ‘least representative’. Participants were asked to talk through their process of ranking, so that we could record qualitative information about the gaps between criteria and their reasoning for each criterions position. A final set of seven was decided upon by the authors that was judged to best balance the different interests of all stakeholder groups.

### 2.3 Step 3: MCDA weights elicitation survey

Using the final set of criteria, a survey was designed using Qualtrics (25) to collect relative numeric weights of the criteria from a range of stakeholders using a technique referred to as Swing weighting. Two rounds of pilot testing of the survey were undertaken to ensure the survey was clear in its requirements and could be completed in a reasonable time frame. Using the results from pilot testing a finalised survey was created. Full details of the survey can be found in Additional File 1. The first four questions were all multiple choice and asked for the respondent’s involvement in clinical trials, whether they have any paid affiliations with the pharmaceutical or biomedical industry, their level of experience designing or running a clinical trial and their country of residence. Participants were then provided with definitions of the seven criteria being assessed and the range of likely values these criteria could take. They were then asked to rank the criteria from most to least important using swing weighting. Swing weighting requires respondents to provide rankings based upon the value gained should a metric improve from its worst possible outcome to its best which provides valuable information on the marginal utility of each metric. Respondents were then asked to provide estimates of weights (0–99) of each metric relative to the top ranked metric which had a weight of 100. The survey was open between 1 May 2020 and 31 July 2020. A different group? of participants were identified through professional networks and discussions with interested stakeholders. Each invited participant was sent an email containing survey details and a link to the survey. Those receiving an email invitation were also invited to forward the survey link to anyone whom they believed might be interested in participating. Consent was requested before participants could complete the survey.

### 2.4 Step 4: Eliciting partial value functions

Each respective criteria in our value framework is converted into a partial value function (PVF) thereby constructing one common ‘value’ scale ranging from 0 to 100. For example, the ‘patient outcomes’ metric is measured in terms of months of survival, which is then transformed into a numerical value between 0 and 100 to construct the PVF. This procedure allows comparison of different criteria measured with different scales. Also, PVFs can then be directly summed to create the overall value measure for a given decision alternative, clinical trials in this instance. For each metric, we established the functional form of each PVF through a series of interviews using the bisection method. The bisection method required that for each metric, respondents were asked at what point within the range of possible values for a given metric, with its worst possible outcome corresponding to a value of 0 and its best possible outcome a value of 100, while the midpoint would be equivalent to a value of 50. Alternatively, at what measure of the metric, was an increase from its worst possible measure to that point, equal in value to an increase from that point to its best possible outcome. All other criteria were held constant throughout.

Interviews to elicit PVFs were conducted with six stakeholder representatives individually. Each interview began with an explanation of the project and why PVFs were required. The bisection method was explained in detail. Participants were then provided with examples and given the opportunity to practice on a simple example. We then explained the definition of each metric and eliciting their PVFs in turn. As participants responded, results were shown graphically to provide the participants with constant feedback. Results were recorded manually throughout.

The PVF for each metric was calculated using the average midpoint of the possible values of a metric, that corresponded to a value of 50 elicited from stakeholders participating in the PVF elicitation interviews. With the midpoint identified, the parameters of a linear function were then calculated to create a straight line from the lowest value point of the metric to the average midpoint. This was repeated from the midpoint to the point of greatest value for each metric. This created ‘bent-stick’ style PVF. These ‘bent-stick’ style PVFs are believed to be more representative of the true PVF than a simple linear PVF while being significantly less complex than generating a higher order function to represent a given PVF. The equations for each PVF can be seen in Additional File 2.

#### 2.5 Step 5**:** Data analysis

Of the completed surveys, responses were separated into one of two categories, concordant or discordant. Concordant responses are those where metric rankings matched the descending order of metric weights. Conversely, discordant responses were those with metric rankings that did not match the descending order of metric weights.

The metric weights provided in the concordant data were standardised so they all fit on the same scale, 0 to 100. The standardisation was carried out using Equation 1 where ‘$w_{i}$’ is the weight provided by the respondent for a given metric.

| $\frac{w_{i}}{\sum_{i=1}^{n} w_{i}}\times100$ |  | (1) |
| --- | --- | --- |

The weights provided by respondents in discordant responses were replaced with a set of weights calculated using the reciprocal of rank formula (Eq. 2) (26). The formula is based on the number of criteria, $n$ and the rank $k$ given by the respondent for that metric (out of $n$), with *j* representing the index value. The sum of the standardised weights for each individual response equalled 100.

| $\frac{\frac{1}{k}}{\sum_{j=1}^{n} \frac{1}{j}}\times100$ |  | (2) |
| --- | --- | --- |

Data from the concordant and, formerly, discordant categories were then combined.

Using the standardised weights calculated for each metric, an average standardised weight was calculated for each metric using the combined discordant and concordant data. Analysis was carried out using R version 4.0.2 (27).

### 2.5.1 Development of an online Shiny app. A publicly available Shiny web app was developed to facilitate the implementation and further development, of the MCDA tool using the results of our study. The Shiny app allows users to manually input or upload a CSV file containing data on the clinical trial criteria. The Shiny web app applies the PVFs and weightings to the data and aggregates the results to produce a single value measure for each trial. The web app can be viewed at Gillett et al., 2020 (28).

### 2.5.2 Validation of the decision tool. Data from six previously completed cancer clinical trials were used as a proof-of-concept of the decision tool. These trials were selected for inclusion as they covered a recent range of years, were relatively representative of cancer clinical trials and mostly had the required information available to be useful. The value of each trial was calculated using the standardised weights and the PVF as programmed in the online tool. Data pertaining to our criteria of interest were collected from the paper and, where information for a specific criterion was not available, it was filled in with a median value or a value from a similar trial as a substitute. The aggregate trial value was calculated for each trial by summing the inputs for each metric, adjusted by the survey derived weights. The results were plotted and highlight the total trial value as well as the respective contributions of each metric.

### A sensitivity analysis was performed on the metric weights comparing outcomes if the combined concordant and discordant data is used, as reported above, or only the concordant data. These results are outlined in Additional File 3.

Table 1

| **Trial** | **Unmet need** | **Size of target population** | **Access to trials** | **Patient outcomes** | **Total trial cost** | **Academic impact** | **Use of trial results** | **Aggregate value** |
| --- | --- | --- | --- | --- | --- | --- | --- | --- |
| Example A (Hammel et al., 2016)(29) | 8.0 | 12.0 | 55.0 | 1.3 | 40.5 | 440.0 | Informed research | 45.81 |
| Example B (Hammel et al., 2016) (29) | 8.0 | 222.0 | 55.0 | 1.7 | 55.0 | 440.0 | Granted regulatory approval | 57.31 |
| Example C (Rombouts et al., 2016) (30) | 35.0 | 12.0 | 55.0 | 14.8 | 13.0 | 31.0 | Informed policy | 51.83 |
| Example D (Yang et al., 2011) (31) | 32.0 | 8.1 | 55.0 | 4.5 | 40.5 | 508.0 | Informed research | 40.61 |
| Example E (Sledge et al., 2020) (32) | 91.0 | 65.0 | 55.0 | 9.4 | 27.0 | 69.0 | Unused | 24.31 |
| Example F (Shroff., 2019) (33) | 24.0 | 7.8 | 55.0 | 7.5 | 40.5 | 40.0 | Unused | 32.19 |
|  |  |  |  |  |  |  |  |  |
|  |  |  |  |  |  |  |  |  |
|  |  |  |  |  |  |  |  |  |
| The five clinical trials (A and B are the same trial but assessed two separate treatment regimens and thus are included separately), the data corresponding to the criteria of interest and their final calculated aggregate value. | | | | | | | | |

## **3 Results**

### 3.1 Final set of criteria

The following seven criteria were selected: unmet need, size of target population, eligible participants can access the trial, patient outcomes, total trial cost, academic impact and use of trial results (Table 2).

Table 2

| **Metric** | **Definition** |
| --- | --- |
| Unmet need | The trial addresses a problem either without a solution or a very poor solution. This could be a rare disease with no treatment options and poor survival. The 5-year survival rates of particular cancers can range from 85% (Good treatment options and therefore there is little to be learnt from another trial) to 18% (There are poor treatment options and thus research in this area will likely be very beneficial or impactful). |
| Size of target population | The burden or prevalence of the target disease the trial seeks to address. A rare disease may only affect 0.2 people / 100,000 while a common disease may affect 1000 people / 100,000. |
| Eligible participants can access the trial (access to trials) | Eligible patients have equal opportunity to enrol in a clinical trial regardless of their geographic location and its associated limitations. Possible responses range from 1 to 5. With 1 = Less than 20% of eligible patients have access to a trial due to geographic limitations and 5 = 100% of eligible patients have access to a trial. |
| Patient outcomes | The increase in overall survival for patients. From a 3 month increase, to 3 years additional survival. |
| Total trial  cost | The total cost of running the trial to completion. This ranges between 105 million AUD (expensive) to 3 million AUD (least expensive). |
| Academic impact | The number of citations the primary publication of trial results receives in the academic and clinical literature. This could range from 10 to 1000 citations. |
| Use of trial results | Whether the results of the trial directly influenced future directions of the research. There are four options: 1) No use of results; 2) informing research decisions such as continuation to another phase, e.g. phase I to II; 3) granting of regulatory approval e.g. FDA or PBS approval; 4) was used to inform policy. The trial results in order of increasing value are, 1) no use of results, 2) informed research decisions, 3) granted regulatory approval, 4) informed policy. |
|  |  |
|  |  |
| The final seven criteria selected for inclusion in the survey, their definitions and possible range of values. | |

### 3.2 Weight elicitation: survey responses and metric ranks

There were 157 unique consenting responses to the survey. Of those, 80 (51%) answered all the required questions. Of the participants who answered all required questions, 43 (53.8%) provided concordant responses and 37 (46.3%) provided discordant responses. Detailed characteristics of the respondents are given in Table 3, broken down by concordant, discordant and all completed responses categories. The average standardised weights for each of the criteria using the combined data can be seen in Table 4. Unmet need had the highest average weight followed by patient outcomes. Use of trial results, size of target population and eligible participants can access the trial filled the next three positions and total trial cost and academic impact were sixth and seventh respectively. The proportion of votes each metric received for each ranking position can be seen in Figure 1. The descending order of the proportion of Rank 1 votes received by each metric is, unmet need (43.8%), patient outcomes (25%), use of trial results (13.8%), size of target population (8.8%) and access to trials (8.8%) were equal, followed by academic impact (0%) and total trial cost (0%) with no Rank 1 votes.

Table 3

|  | **Participant Background** | |  |
| --- | --- | --- | --- |
| **Characteristic** | **Concordant data (%)** | **Discordant data (%)** | **Total (%)** |
| Paid affiliation |  |  |  |
| Yes | 3 (7) | 4 (11) | 7 (9) |
| No | 40 (93) | 33 (89) | 73 (91) |
| Trial experience |  |  |  |
| No experience | 8 (18) | 12 (32) | 20 (25) |
| Less than 10 years experience | 14 (33) | 14 (38) | 28 (35) |
| 10 years or greater experience | 18 (42) | 11 (30) | 29 (36) |
| Other | 3 (7) | 0 (0) | 3 (4) |
| Background |  |  |  |
| Health economist | 3 (7) | 2 (5) | 5 (6) |
| Health professional | 9 (21) | 16 (44) | 25 (31) |
| Patient / consumer | 7 (16) | 8 (22) | 15 (19) |
| Scientist | 5 (12) | 2 (5) | 7 (8) |
| Statistician | 11 (25) | 3 (8) | 14 (18) |
| CRA/CRO | 6 (14) | 2 (5) | 8 (10) |
| Other | 2 (5) | 4 (11) | 6 (8) |
|  |  |  |  |
|  |  |  |  |
| Participant characteristics of respondents, including whether respondents had, in the last three years, any paid affiliations with pharmaceutical, medical device or diagnostic companies. Trial experience refers to whether professionals had any experience designing or running a clinical trial, participation in a clinical trial as a patient was not included in this category. Background of the respondent refers to the reason for their interest or involvement in clinical trials. The patient/consumer category included all current and former trial patients, current or former cancer patients not involved in a clinical trial, parents, guardians or caregivers of children currently or formerly involved in clinical trials and consumer or patient advocates in general. CRA/CRO is a broad term referring to any respondent who identified themselves as being a clinical research associate, working for a clinical research organisation, a clinical trial manager, study coordinator or related position. All respondents resided in Australia at the time of completing the survey. | | | |

Table 4

| **Metric** | **Average Standardised Weight** |
| --- | --- |
| Unmet need | 24.4 |
| Patient outcomes | 21.2 |
| Use of trial results | 14.4 |
| Size of target population | 13.2 |
| Eligible participants can access the trial (access to trials) | 12.4 |
| Total trial cost | 7.4 |
| Academic impact | 7.2 |
|  |  |
|  |  |
| The mean standardised weights for all respondents. | |


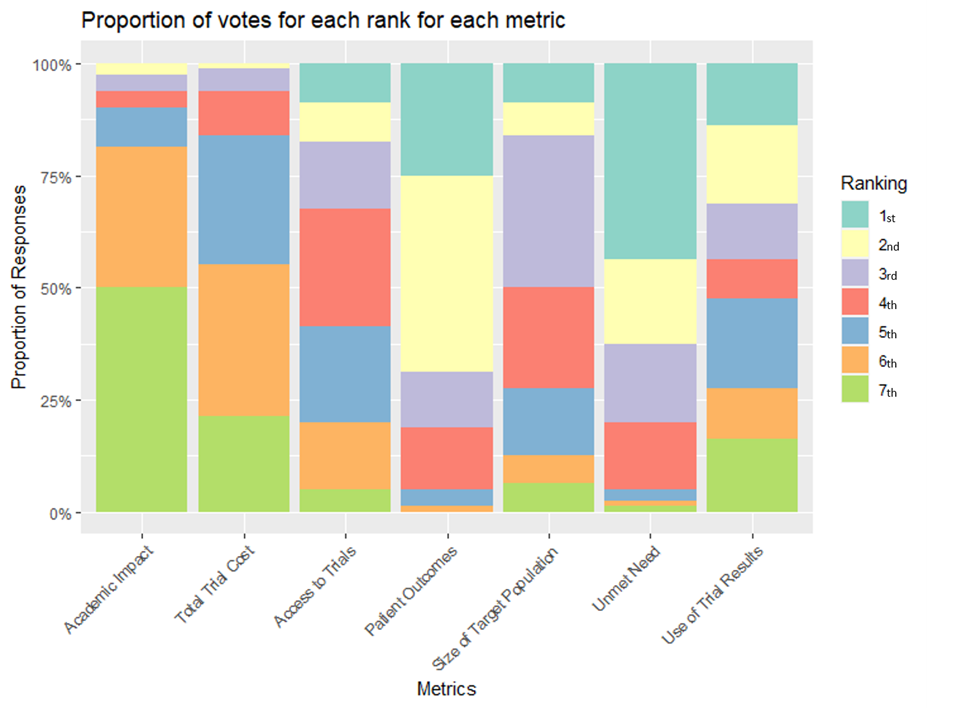


**Figure 1** Proportion of responses each metric received for a specific ranking position

### 3.3 Application to real-world cancer clinical trials

Six cancer clinical trials were evaluated using the MCDA tool implemented via the Shiny web app. The results are displayed in Table 1. Trials were selected to cover a wide range of the included criteria. The trial with the greatest value of the example group was Hammel B 2016 with a score of 57.31, primarily due to its high ‘unmet need’. In descending order of trial value was Rombouts 2016 (51.83), Hammel A 2016 (45.81), Yang 2011 (40.61), Shroff 2019 (32.19) and Sledge 2020 (24.31).

For the six example trials, the most impactful metric driving changes in overall value is ‘unmet need’ as it contributed the most value to four of the six trials assessed.

## **4 Discussion**

### 4.1 Outcomes

The results of this work demonstrate the feasibility of using a decision analytical approach to retrospectively value clinical trials. We believe that our MCDA decision tool is an important step toward improving the process of clinical trial prioritisation. Further, we have provided a convincing proof-of-concept through our use of real-world trial data. The same principles may be used by funders or clinical trial units to prioritise new trials, yet this requires prospective validation and reliable estimation of the trial criterions’ performance prior to trial completion. For estimated clinical outcomes, this may be a difficult process.

### Position in the field

Due to the novelty of our work, there are no known directly comparable approaches. The closest examples and perhaps the most well-known are the ESMO magnitude of clinical benefits scale (6) the ASCO value framework (5) and using value of information for real time prioritisation decisions (34). The ESMO framework attempts to balance the benefits of a treatment against any side effects. The ASCO framework sets out to balance treatment benefits against cost. Utilisation of value of information allowed for prospective prioritisation of phase II/III cancer clinical trials. Each respective tool has their place but, in comparison to our decision tool, each considers a clinical trial value in a highly restricted way. Additionally, each of the frameworks are focused on latter phase trials, while our tool is potentially much more broadly applicable.

Due to the dearth of directly comparable decision tools or frameworks, it is difficult to draw comparisons between the resultant metric rankings and those of any other tool. Nonetheless the results of the survey provided interesting results with the average standardised weights of each metric forming three seemingly distinct tiers with similar values. Tier 1 consisted of ‘unmet need’ and ‘patient outcomes’, tier 2 ‘access to trials’, ‘size of target population’, ‘use of trial results’ and tier 3 with ‘academic impact’ and ‘total trial cost’. Figure 1 may provide some clarity as to this outcome as ‘unmet need’ for example received the greatest number of first place rankings but also the greatest number of third place rankings. ‘Patient outcomes’ received the second most first place rankings but the most second place rankings. The corresponding weights attributed to each metric position by respondents may have resulted in a balancing of the different rankings resulting in the similar weights. This could also extend to the remaining criteria and highlights the heterogeneity of preferences the stakeholders held. It could be argued that the variation in rankings is due to respondent fatigue completing a lengthy survey or confusion over the process. Within the field though, the use of seven criteria is very close to the average of 8.2 criteria across the MCDA literature (35) and thus we believe this is most likely the outcome from surveying a diverse group of stakeholders.

### Strengths and weaknesses

Our study is the first of its kind and not without limitations. A key difficulty that we encountered was the varying levels of clinical trial knowledge among participants. By presenting the survey to as many interested groups as possible we included participants who had only a limited familiarity with clinical trials. This was highlighted by the fact that a greater proportion of respondents identifying as patients/consumer advocates/family members of patients started the survey and then failed to complete it. This pattern of participant dropout may have altered the results to some extent; however, we believe our results are robust to this effect, but this has not been evaluated.

The broad inclusion of interested stakeholders is also a key strength of our method as it explicitly incorporates the subjective preferences of a diverse group of stakeholders. While it could be argued further additional stakeholder groups should have been included, we nonetheless captured the majority of possible stakeholders. Incorporating input from this diverse group of stakeholders enabled a much more holistic assessment of trial value. It would be straightforward to adjust the criteria used to represent the values of one specific stakeholder group, but we believe the strength of our methods comes from the inclusion of multiple stakeholder groups. Whether using preferences from diverse stakeholders or a single group, the use of our decision tool provides a transparent first step towards methods to screen prospective trials at very low cost.

Furthermore, although our MCDA decision tool is developed in the context of cancer clinical trials, it could be further extended to trials covering other diseases through improvement of the ‘patient outcomes’ metric. Currently, only trials reporting a difference in overall survival between groups can be assessed. By extending the metric to enable trials that report, for example, progression-free survival, quality adjusted life years or toxicity, a more varied range of trial types could be assessed. Inclusion of other clinical endpoints is technically feasible, such as symptom scores for example, but it would require re-assessment of the weights for each metric.

### 4.4 Next steps

As the tool is in its early stages, further development is required before it’ll be implemented for prospectively valuing clinical trials. Regardless of this, there remains key lessons taken from its development that could be used to improve the process of clinical trial portfolio management that exists today, particularly stakeholder engagement. Processes by which trials are selected by clinical trial units and the individuals involved in the decision vary based upon the needs and priorities of a given community or institution (36). The inclusion of at least a patient or consumer advocate in the trial selection process stands to improve the likelihood that trials that don’t provide meaningful benefit to patients, whether that be strictly in terms of improved outcomes or reduced side effects leading to better quality of life, are rejected in favour of those that do. Furthermore, greater patient engagement should it identify undesirable trial proposals before their launch, may also reduce overall costs by preventing their launch.

## **5 Conclusions**

This study has demonstrated the feasibility of a broadly applicable tool for assigning value to clinical trials across a range of criteria. It is a transparent and objective tool by which to evaluate clinical trials for the purposes of prioritisation. Our hope is that the tool is used by decision makers to improve allocation of scarce medical research resources and ultimately improve patient outcomes.

## **References**

1. Sculpher MJ, Claxton K, Drummond M, McCabe C. Whither trial-based economic evaluation for health care decision making? Health Econ. 2006 Jul;15(7).

2. DiMasi JA, Grabowski HG, Hansen RW. Innovation in the pharmaceutical industry: New estimates of R&D costs. J Health Econ. 2016;47:20–33.

3. Sullivan F&. Australia : Preferred Destination for Early Phase Clinical Trials. 2016.

4. Heath A, Myriam Hunink MG, Krijkamp E, Pechlivanoglou P. Prioritisation and design of clinical trials. Eur J Epidemiol. 2021 Nov 6;36(11):1111–21.

5. Schnipper LE, Davidson NE, Wollins DS, Blayney DW, Dicker AP, Ganz PA, et al. Updating the American society of clinical oncology value framework: Revisions and reflections in response to comments received. Journal of Clinical Oncology. 2016;34(24):2925–33.

6. Cherny NI, Dafni U, Bogaerts J, Latino NJ, Pentheroudakis G, Douillard JY, et al. ESMO-Magnitude of Clinical Benefit Scale version 1.1. Annals of Oncology. 2017;28(10):2340–66.

7. del Paggio JC, Sullivan R, Schrag D, Hopman WM, Azariah B, Pramesh CS, et al. Delivery of meaningful cancer care: a retrospective cohort study assessing cost and benefit with the ASCO and ESMO frameworks. Lancet Oncol. 2017 Jul;18(7).

8. Davis C, Naci H, Gurpinar E, Poplavska E, Pinto A, Aggarwal A. Availability of evidence of benefits on overall survival and quality of life of cancer drugs approved by European Medicines Agency: retrospective cohort study of drug approvals 2009-13. BMJ. 2017 Oct 4;

9. Vivot A, Jacot J, Zeitoun JD, Ravaud P, Crequit P, Porcher R. Clinical benefit, price and approval characteristics of FDA-approved new drugs for treating advanced solid cancer, 2000-2015. Ann Oncol. 2017 May 1;28(5):1111–6.

10. Higgins JPT, Altman DG, Gøtzsche PC, Jüni P, Moher D, Oxman AD, et al. The Cochrane Collaboration’s tool for assessing risk of bias in randomised trials. BMJ (Online). 2011;

11. Jadad A, Moore A, Carrol D, Jenkinson C, Reynolds J, Gavghan D, et al. Assessing the Quality of Reports of Randomized Clinical Trials: Is blinding Necessary. Control Clin Trials. 1996;1–12.

12. Cho MK, Bero L a. Instruments for Assessing the Quality of Drug Studies Published in the Medical Literature. JAMA. 2010;14–7.

13. Nuromohamed M, Rosendaal F, Buller H, Dekker E, Hommes D, Vandenbroucke J, et al. Low-molecular weight heparin versus standard heparin in general and other orthopaedic surgery: a meta-analysis. The Lancet. 1992;10(3):131–4.

14. Verhagen A, de Vet H, de Bie R, Kessels A, Boers M, Bouter L, et al. The Delphi List: A Criteria List for Quality Assessment of Randomized Clinical Trials for Conducting Systematic Reviews Developed by Delphi Consensus. J Clin Epidemiol. 1998;51(12):1235–41.

15. Thokala P, Devlin N, Marsh K, Baltussen R, Boysen M, Kalo Z, et al. Multiple criteria decision analysis for health care decision making - An introduction: Report 1 of the ISPOR MCDA Emerging Good Practices Task Force. Value in Health. 2016;

16. Hummel JM, Bridges JFP, IJzerman MJ. Group decision making with the analytic hierarchy process in benefit-risk assessment: A tutorial. Patient. 2014.

17. Oliveira MD, Mataloto I, Kanavos P. Multi-criteria decision analysis for health technology assessment: addressing methodological challenges to improve the state of the art. European Journal of Health Economics. 2019;

18. Adunlin G, Diaby V, Montero AJ, Xiao H. Multicriteria decision analysis in oncology. Health Expectations. 2015;

19. Marsh K, Ijzerman M, Thokala P, Baltussen R, Boysen M, Kaló Z, et al. Multiple Criteria Decision Analysis for Health Care Decision Making - Emerging Good Practices: Report 2 of the ISPOR MCDA Emerging Good Practices Task Force. Value in Health. 2016 Mar 1;19(2):125–37.

20. Chapple CR, Mironska E, Wagg A, Milsom I, Diaz DC, Koelbl H, et al. Multicriteria Decision Analysis Applied to the Clinical Use of Pharmacotherapy for Overactive Bladder Symptom Complex. Eur Urol Focus. 2020 May;6(3):522–30.

21. Ahuja S, Phillips L, Smartt C, Khalid S, Coldham T, Fischer L, et al. What interventions should we implement in England’s mental health services? The mental health implementation network (MHIN) mixed-methods approach to rapid prioritisation. Frontiers in Health Services. 2023 Aug 11;3.

22. Guthrie S, Krapels J, Lichten C, Wooding S. 100 Metrics to Assess and Communicate the Value of Biomedical Research: An Ideas Book. RAND Corporation; 2016.

23. Deshpande A, Guthrie S, Cox C, Bertscher A. Development of metrics to measure the impact of Tommy’s work. RAND Corporation; 2018.

24. Poll Everywhere. https://www.polleverywhere.com/. 2019. Poll Everywhere.

25. Qualtrics. Qualtrics. Provo, Utah, USA: Qualtrics; 2020.

26. Barron FH, Barrett BE. Decision quality using ranked attribute weights. Manage Sci. 1996;42(11):1515–23.

27. R Core Team. R: A language and environment for  statistical computing. vienna, Austria: R Foundation for Statistical Computing; 2020.

28. Gillett P, Mahar RK, Tran NR, Rosenthal M, IJzerman M. An R Shiny app retrospectively valuing clinical trials using MCDA [Internet]. 2020 [cited 2022 Jun 22]. Available from: https://cancerhealthservices.shinyapps.io/ValuingClinicalTrials/?_ga=2.1086130.223167595.1595799553-25929989.1593641240

29. Hammel P, Huguet F, van Laethem JL, Goldstein D, Glimelius B, Artru P, et al. Effect of Chemoradiotherapy vs Chemotherapy on Survival in Patients With Locally Advanced Pancreatic Cancer Controlled After 4 Months of Gemcitabine With or Without Erlotinib. JAMA. 2016 May 3;315(17):1844.

30. Rombouts SJ, Mungroop TH, Heilmann MN, van Laarhoven HW, Busch OR, Molenaar IQ, et al. FOLFIRINOX in Locally Advanced and Metastatic Pancreatic Cancer: A Single Centre Cohort Study. J Cancer. 2016;7(13):1861–6.

31. Yang XJ, Huang CQ, Suo T, Mei LJ, Yang GL, Cheng FL, et al. Cytoreductive Surgery and Hyperthermic Intraperitoneal Chemotherapy Improves Survival of Patients with Peritoneal Carcinomatosis from Gastric Cancer: Final Results of a Phase III Randomized Clinical Trial. Ann Surg Oncol. 2011 Jun 23;18(6):1575–81.

32. Sledge GW, Toi M, Neven P, Sohn J, Inoue K, Pivot X, et al. The Effect of Abemaciclib Plus Fulvestrant on Overall Survival in Hormone Receptor–Positive, ERBB2-Negative Breast Cancer That Progressed on Endocrine Therapy—MONARCH 2. JAMA Oncol. 2020 Jan 1;6(1):116.

33. Shroff RT, Javle MM, Xiao L, Kaseb AO, Varadhachary GR, Wolff RA, et al. Gemcitabine, Cisplatin, and nab-Paclitaxel for the Treatment of Advanced Biliary Tract Cancers. JAMA Oncol. 2019 Jun 1;5(6):824.

34. Bennette CS, Veenstra DL, Basu A, Baker LH, Ramsey SD, Carlson JJ. Development and Evaluation of an Approach to Using Value of Information Analyses for Real-Time Prioritization Decisions Within SWOG, a Large Cancer Clinical Trials Cooperative Group. Medical Decision Making. 2016 Jul 1;36(5):641–51.

35. Marsh K, Lanitis T, Neasham D, Orfanos P, Caro J. Assessing the Value of Healthcare Interventions Using Multi-Criteria Decision Analysis: A Review of the Literature. Pharmacoeconomics. 2014 Apr 7;32(4):345–65.

36. Identifying and selecting a clinical trial for your practice. J Oncol Pract. 2008 Jan;4(1):27–8.
